# Predicting the Multisensory Consequences of One’s Own Action: BOLD Suppression in Auditory and Visual Cortices

**DOI:** 10.1371/journal.pone.0169131

**Published:** 2017-01-06

**Authors:** Benjamin Straube, Bianca M. van Kemenade, B. Ezgi Arikan, Katja Fiehler, Dirk T. Leube, Laurence R. Harris, Tilo Kircher

**Affiliations:** 1 Philipps-University Marburg, Department of Psychiatry and Psychotherapy, Marburg, Germany; 2 Justus-Liebig University, Department of Experimental Psychology, Giessen, Germany; 3 AWO Centre of Psychiatry Halle, Clinic for Psychiatry and Psychotherapy, Halle, Germany; 4 York University, Centre for Vision Research and Department of Psychology, Ontario, Canada; Harvard Medical School, UNITED STATES

## Abstract

Predictive mechanisms are essential to successfully interact with the environment and to compensate for delays in the transmission of neural signals. However, whether and how we predict *multisensory* action outcomes remains largely unknown. Here we investigated the existence of multisensory predictive mechanisms in a context where actions have outcomes in different modalities. During fMRI data acquisition auditory, visual and auditory-visual stimuli were presented in active and passive conditions. In the active condition, a self-initiated button press elicited the stimuli with variable short delays (0-417ms) between action and outcome, and participants had to detect the presence of a delay for auditory or visual outcome (task modality). In the passive condition, stimuli appeared automatically, and participants had to detect the number of stimulus modalities (unimodal/bimodal). For action consequences compared to identical but unpredictable control stimuli we observed *suppression* of the blood oxygen level depended (BOLD) response in a broad network including bilateral auditory and visual cortices. This effect was independent of task modality or stimulus modality and strongest for trials where no delay was detected (undetected<detected). In bimodal vs. unimodal conditions we found activation differences in the left cerebellum for detected vs. undetected trials and an increased cerebellar-sensory cortex connectivity. Thus, action-related predictive mechanisms lead to BOLD suppression in multiple sensory brain regions. These findings support the hypothesis of multisensory predictive mechanisms, which are probably conducted in the left cerebellum.

## Introduction

Perceiving one`s own actions and related sensory action consequences is essential to successfully interact with the environment. One’s own action consequences are highly predictable and therefore require less sensory resources than the processing of unpredictable external events. Predictive mechanisms allow us to anticipate the future state of both the environment and ourselves in order to compensate for delays in the transmission of neural signals and distinguish external events from the sensory consequences of our own actions [1]. Predictions are found at different levels of processing, from simple eye movements to complex motor acts or language processing, and they have even been identified as one of the defining functions of the human brain [2]. Efference copies [3, 4] of motor outputs can be used to predict re-afferent sensory feedback (see [5], for a review). They modulate the response properties of the corresponding sensory cortex and prepare it for re-afferent stimuli [5]. This is known as the forward model (e.g., [6, 7]) which presumably increases the efficiency of attention and cognitive processing by preventing the central nervous system from wasting neural resources on irrelevant sensory stimuli [1]. This process also allows sensory re-afferents from motor outputs to be recognised as the self-generated result of an action. So far, ‘predictive mechanisms’ on a neural level have only been studied for single modalities such as responses to tactile [8–10], visual [11–16] or auditory stimuli [17]. Since real-world actions usually stimulate several senses simultaneously (e.g., seeing, feeling and hearing my own hands clapping), the question arises whether and how we predict *multisensory* action outcomes.

Multisensory processing mechanisms have often been related to facilitation in a variety of tasks [18]. In these cases it has been assumed that events in a modulating modality (e.g., a sound) may render a particular space (and/or time) salient for another modality (e.g., a visual stimulus), to facilitate modality-specific processing for that time or place in the latter modality ([19–22]; see [18], for a review). However, the challenge for the brain is to connect the different kind of information in a suitable way, especially because in an early stage different unisensory brain regions, e.g. auditory and visual cortices, are in charge of processing incoming information. The cerebellum is a good candidate brain region which might contribute to the prediction of multisensory action outcomes, since it is relevant for visual and auditory processing, timing, perceptual sequencing and predictive processing and is functional connected to visual and auditory sensory cortices (see [23] for an overview). Despite the fact that first behavioral evidence suggests the existence of multisensory predictive mechanisms for auditory-visual action consequences [24], the neural correlates of these processes remain unknown. Therefore, the current study focused on the neural processing of multisensory consequences of one’s own action.

The principles of action prediction have been investigated with paradigms probing anticipated action effects. Behaviorally, it has been shown that self-generated stimuli are perceived as less intense compared to externally generated stimuli, a phenomenon known as sensory attenuation [6]. Sensory attenuation has been demonstrated in the somatosensory [25], auditory [26] and visual domains ([27, 28]; see [29] for a review). These behavioral studies have been complemented by electrophysiological correlates of anticipated action effects (e.g., [25, 30–36]). Studies using fMRI suggest an involvement of the cerebellum in predicting action outcomes [9, 14, 16, 32] and provide evidence for BOLD suppression for predictable compared to unpredictable (e.g., delayed) action outcomes in visual [11, 15, 16], auditory [17] and somatosensory [8–10, 37–39] brain regions. However, up till now, sensory suppression at neural level has only been studied for individual modalities separately. Thus, whether actions with potential consequences in multiple modalities lead to BOLD suppression in multiple sensory processing areas in the brain is unknown.

Various tasks have been used to study predictive mechanisms and related sensory suppression at a neural level. These include looking at active action conditions in which the consequences are remapped to new spatial (e.g., real vs. rotated feedback of the hand [15]), temporal (e.g., delayed feedback [11, 14, 17, 32, 40, 41]) or unpredictable (e.g., passive movement or other control conditions [8–10]) outcomes. Delay detection tasks, in which a short interval between one’s own action and the resulting perceptual consequences has to be detected, have several advantages for studying predictive mechanisms [11, 14, 17, 40]: because they 1) focus participants’ attention on the perceptual consequences of an action, 2) make it possible to compare subjectively instantaneous trials (in which reafferent feedback matches the prediction) with delayed trials (in which feedback is unpredictable), and 3) can be applied to action outcomes in multiple modalities. Up to now, delay detection tasks have only been applied to single modalities in imaging studies. However, on behavioral level we successfully applied the delay detection task to multiple modalities and found evidence for bimodal facilitation for the detection of delays [24].

In the current study, the neural correlates of predicting multisensory action consequences were investigated using fMRI, by adopting the basic design of the behavioural study [24]. In an active condition, self-initiated hand movements (button presses) elicited the presentation of stimuli in the visual and auditory modality with variable short delays (0–417 ms) between the action and its outcome. In a passive control condition, the same auditory, visual and auditory-visual stimuli were presented, unconnected to the participant’s actions (participants did not move) and consequently unpredictable. In the active condition, participants had to detect delays between action and feedback. Thus, although technically there were more delayed trials than non-delayed trials, the participants’ default temporal prediction was set to a delay of 0ms, by explicitly instructing participants to detect sensory information that deviated temporally from this action-based expectation. In the passive condition, participants only had to report whether they saw a unimodal or bimodal stimulus. Since real life actions (e.g., hand clapping or knocking on a door) usually have multisensory consequences we hypothesized that both multisensory and unisensory consequences would be predicted (see [24]) and therefore the corresponding neural signals would be suppressed compared to when the same stimuli were unpredictable. Thus, compared to studies focussing on single modalities and related suppression in respective (uni-)sensory brain regions, we expected BOLD suppression in multiple sensory brain regions (e.g., auditory and visual cortices). Furthermore, we expected that BOLD suppression in auditory and visual sensory cortices would be independent of feedback modality, since visual, auditory and audio-visual consequences were equally predictable. Finally, we expected the strongest suppression effects to occur in trials that were perceived as simultaneous with the action, as for these trials the action consequences occurred as predicted/ in line with the default expectation (i.e. no violation of the temporal contiguity could be detected).

## Methods

### Participants

21 healthy, right-handed (Edinburgh Handedness Inventory [42]) participants with normal or corrected-to-normal vision took part in the experiment (8 males, age range 19–30, mean age 24.9 years). One participant had to be excluded from the fMRI analysis because of excessive movement, resulting in a sample of twenty participants (8 males, age range 19–30, mean age 25.1 years). For the subsequent analysis comparing detected vs. undetected delays, three further subjects had to be excluded because of their small number of trials per experimental run (see *fMRI data analysis*), resulting in a final group of seventeen participants (7 males, age range 19–30, mean age 25 years) for the second analysis. The study was approved by the local ethics committee of the medical faculty of the Philipps-University Marburg, Germany (https://www.uni-marburg.de/fb20/ethikkommission; registration number: 123/13) in accordance with the Declaration of Helsinki. Written informed consent has been obtained from all participants.

### Stimuli and procedure

During fMRI data acquisition participants wore headphones (MR-Confon Optimel, Magdeburg, Germany) through which auditory stimuli were delivered in the form of a pure-tone 250Hz beep (presented for 1 second). The visual stimulus was a black dot (1.5° visual angle), presented (for 1 second) centrally on a medium grey background on a computer screen (refresh rate 60 Hz) positioned behind the scanner. The screen was viewed by the participants in an appropriately angled mirror. Participants placed their right hand on a button pad, with their right index finger touching the button. The button pad was fixed on their right leg. The left index and middle finger were placed on two buttons of a separate button pad located and fixed on the left leg. Stimuli were presented using Octave and the Psychtoolbox [43].

**Fig 1:**
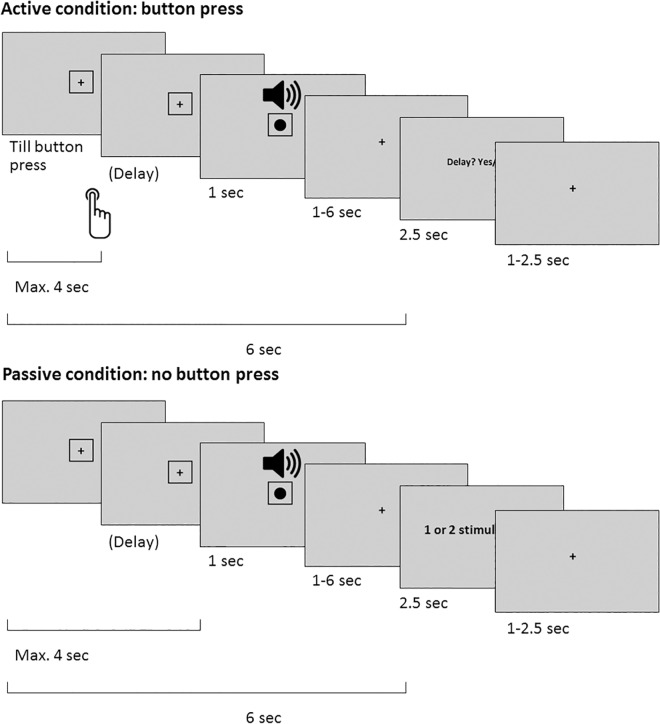
An example of a bimodal trial. In the active condition (top) participants had to wait with their button press until the cue appeared, and could take as much time as they wanted (max. 4 seconds). After a variable delay, unimodal or bimodal stimuli were presented. Participants had to report whether they detected a delay between their button press and the stimulus of the task modality. In the passive condition (bottom), an identical trial structure was used. However, no button press was performed by the participants and they had just to report whether they perceived one or two stimuli.

The general paradigm (Fig 1) has been adapted from a previous behavioral study [24]. However, due to technical reasons an externally-controlled (passive) moving button could not be included in the current imaging study. The participants had to perform button presses with their right index finger, which would elicit the appearance of either the dot on the screen, or the tone, or both. The stimuli were presented either at the time of the button press, or with a variable delay. The participants’ task was to detect the presence of a delay between their button press and the presented stimuli. They answered ‘Yes, there was a delay’ by pressing a button with their left middle finger, or ‘No, there was no delay’ by pressing a button with their left index finger. Participants always had to report the delays in only one modality, referred to as ‘task modality’ in this article. Thus, in bimodal trials participants only had to report whether they detected a delay between their action and the target stimulus, i.e. the stimulus in the other modality (referred to as ‘task-irrelevant modality’) was not important for the task. Participants were instructed at the start of each mini-block (12 trials) about the target stimuli (task modality) via written instruction (auditory task or visual task). There were 5 mini-blocks in each run (in total 60 trials per run). The task order was either visual–auditory–passive–visual–auditory, or auditory–visual–passive–auditory–visual. In active trials the delay between action and stimulus was one of the six predefined delays (0, 83, 167, 250, 333, or 417 ms, presented in frames (0, 5, 10, 15, 20, or 25 frames)). In bimodal trials, the two components of the stimulus were always presented together. Unimodal and bimodal trials were randomized within each mini-block.

The procedure during a trial was as follows (see Fig 1). Each trial started with the presentation of a fixation cross presented for a variable intertrial interval (1, 1.5, or 2 seconds), after which a cue appeared in the form of the outline of a square (3.2° visual angle), surrounding the fixation cross.

In the *active condition*, the cue indicated that from now on, participants could press the button with their right index finger, which triggered the unimodal or bimodal stimulus after a delay of 0-417ms. The participants were instructed to perform button presses at their own pace in a fixed time window up to four seconds after the cue onset. The visual stimulus appeared at the location of the fixation cross, thus obscuring it. For unimodal auditory trials the fixation cross remained visible during the presentation of the tone. The cue and stimuli disappeared at the same time. Subsequent to the offset of the stimuli and cue, there was a variable interval with the fixation cross before the question ‘Delay? Yes/No’ was presented on the screen, after a fixed period of six seconds after cue onset.

In the *passive condition*, participants were instructed not to press the button when they saw the cue, but to just observe and listen to the presented stimuli. In these trials, the stimuli were presented automatically after a variable delay (0.5–3.5 seconds) followed by a fixation cross. After a fixed period of six seconds after cue onset, participants had to judge whether one or two stimuli had been presented. They answered the question “Two stimuli? Yes/no” with their left middle finger for “Yes, there were two stimuli”, or left index finger for “No, there was only one stimulus”. We introduced this bimodal detection task in order to have a similar trial structure and decision processes in the active and passive conditions. Furthermore, this task was easier than the delay detection task in the active condition. Therefore, it was unlikely that the expected suppression effects in active trials (passive>active) were confounded by an increased task demand in the passive condition.

Participants were instructed to be as accurate as possible, but were not required to be as fast as possible. They were given up to 2.5 seconds for their answer. Then the next trial started irrespective of the answer. Missing trials were not repeated to maintain a fixed data acquisition procedure for all experimental runs and participants.

Prior to the fMRI experiment, participants were familiarized with the paradigm in a behavioural training outside the scanner. First, they could press the button several times to experience delayed (417 ms) and undelayed feedback. Then, to become familiar with the paradigm, they completed one run, with the same procedure and number of trials (60 trials) as the fMRI experiment in which they were given feedback about their performance (correct or incorrect). Then, they completed two more runs without feedback. Only subjects with a performance higher than 50% correct were invited to the fMRI study. All 21 of the original sample met this criterion.

The fMRI experiment comprised 300 trials in total: we presented 10 trials for each delay, thus leading to 60 unimodal visual trials (VU), 60 unimodal auditory trials (AU), 60 bimodal visual trials (VB) and 60 bimodal auditory trials (AB). Furthermore, unimodal and bimodal passive control conditions were presented: 20 trials visual unimodal (CV), 20 trials auditory unimodal (CA) and 20 trials bimodal (CB). Stimuli were presented in a rapid event-related fMRI design which was divided into five runs, each comprising 60 trials with 5 mini-blocks.

### Analysis of the behavioral data

Percent delay responses per condition (VU, AU, VB, AB) were used to compare performance between conditions. Additionally, the average delay per condition (detected: VU-d, AU-d, VB-d, AB-d; undetected: VU-nd, AU-nd, VB-nd, AB-nd) were calculated and compared as pseudo-depended variable (see [44] for a comparable approach). Finally, the button press latencies between conditions were compared and correlated with the respective performance per condition to explore potential relationships and to rule out potential confounds due to differences in button press latencies between conditions.

Repeated-measures ANOVAs were performed using SPSS on the percent delay responses and average delays, which were calculated for each participant individually. In the analysis, unimodal trials were compared to all bimodal trials together. Posthoc t-tests (Bonferroni corrected) were conducted to verify the direction of the effects.

### fMRI data acquisition

MRI data were collected using a Siemens 3 Tesla MR Magnetom Trio Trim scanner. In order to minimize head motion artefacts, participants’ heads were fixed using foam pads.

For each experimental run a total of 396 transversal functional images (echo-planar images, 64 x 64 matrix; 34 slices descending; field of view [FoV] = 230 mm; repetition time [TR] = 1650 ms; echo time [TE] = 30 ms; flip angle = 70°; slice thickness = 4.0 mm, gap size: 15%, and voxel resolution = 3 x 3 x 4.6 mm) that covered the whole brain (incl. cerebellum) and were positioned parallel to the intercommissural line (anterior commissure–posterior commissure) were recorded.

### fMRI data analysis

Magnetic resonance images were analyzed using standard routines of Statistical Parametric Mapping (SPM12; www.fil.ion.ucl.ac.uk) implemented in MATLAB 7.9 (Mathworks, Sherborn, Massachusetts). For data preprocessing, standard realignment, coregistration between structural and functional scans, segmentation, normalisation (Montreal Neurological Institute [MNI] template 2 x 2 x 2 mm) and smoothing (8mm) functions of SPM12 were applied.

For single subject analyses, realignment parameters were included as regressors of no interest to account for movement artifacts. Low frequencies were removed using a high-pass filter with a cut-off period of 128 seconds. For the first set of analyses, the hemodynamic response triggered by each visual, auditory or bimodal stimulus of each condition (VU, AU, VB, AB, VC, AC, BC) was modeled with a canonical HRF. For the second set of analyses, active trials were additionally divided into those where delays were detected (VU-d, AU-d, VB-d, AB-d) and those where delays were not detected (VU-nd, AU-nd, VB-nd, AB-nd) leading to eight conditions. Additionally, button presses were included as single additional condition (not separated for modality) of no interest in the single subject models. Of note, the modulation of button presses had a significant effect on the result pattern, when comparing active vs. passive trials. Therefore, we provide additional information in the results section, when results are highly dependent on the modulation of button presses. Parameter estimates (b) and t-statistic images were calculated for each subject.

At the group level (second level analysis), we first performed a random effects group analysis by entering the parameter estimates for seven conditions (VU, AU, VB, AB, VC, AC, BC) into a flexible factorial analysis. In a second flexible factorial group analysis, contrast images of the active conditions separated for detected and undetected trials were entered (VU-d, AU-d, VB-d, AB-d, VU-nd, AU-nd, VB-nd, AB-nd).

To correct for errors of multiple comparisons, we employed family wise error correction (FWE) implemented in SPM12 at p < 0.05. To avoid type II error, we further explored results at p < 0.001 uncorrected, with a cluster extent of 50 contiguous resampled voxels. This threshold is more liberal than the FWE correction but still exceeds a cluster threshold calculated by monte-carlo simulations (http://www2.bc.edu/?slotnics/scripts.htm; see [45]), which suggested 47 activated continuous voxels at *p* < 0.001 uncorrected are sufficient to correct for multiple comparisons at cluster level (p < .05).

The reported voxel coordinates of activation peaks correspond to the MNI space (ICBM standard). For anatomical localization functional data were referenced to the AAL toolbox [46] and the probabilistic cytoarchitectonic maps [47].

Exploratory connectivity analyses in the form of psychophysiological interaction (PPI) analyses, were conducted to better explain the condition specific association between activation change in auditory and visual cortices and the observed results in the cererebellum, motor cortex and SMA.

#### Contrasts of interest

Following our hypotheses, contrasts of interest focused on sensory suppression as reflected in activation differences between active and control conditions (active action feedback < passive control conditions) as well as subjectively delayed vs. undelayed trials (detected > undetected). Interaction effects of task and feedback modality were calculated to explore specific effects for multisensory processing of action consequences. Finally, correlation analyses with behavioural data were performed to explore the relationship between BOLD suppression and behaviour.

Analyses were structured in two steps. First, all active action feedback conditions (VU, AU, VB, AB) were contrasted with respective control conditions (VC, AC and BC), to test for action-dependent BOLD suppression across conditions (VU<VC, AU<AC, VB<BC and AB<BC). Conjunction analyses (minimum t-statistics; [48]) were applied to test for task- and modality-independent BOLD suppression (VU<VC ∩ AU<AC ∩ VB<BC ∩ AB<BC). In a second step, trials where delays had been detected (VU-d, AU-d, VB-d, AB-d) were separated from trials where delays had not been detected (VU-nd, AU-nd, VB-nd, AB-nd) for each active condition. With this analysis we first tested specifically for BOLD suppression for undetected conditions (detected > undetected) in sensory brain regions (auditory/visual cortices) by applying an inclusive masking procedure using the result pattern of the first analyses (conjunction analysis; see Table 1), then we explored the general neural processes related to the detection of delays (detected>undetected) using whole brain analyses. Finally, interaction analyses were applied to test for effects of task (visual/auditory) and modality (unimodal/bimodal) on the neural processing of action consequences subjectively perceived as delayed compared to those perceived as undelayed conditions (detected/undetected).

**Table 1:** *Processing of action consequences compared to unpredictable control stimuli (conjunction analysis across conditions*: VU<VC ∩ AU<AC ∩ VB<BC ∩ AB<BC).

Anatomical Region			Coordinates				no. Voxels
	Cluster extent	Hem.	*x*	*y*	*z*	*t*-value	
PCG	PRG, IPL	Left	-36	-24	52	13.381	1209
STG	Heschl’s gyrus, RO	Left	-46	-24	8	10.010	891
	STG, Heschl’s gyrus, RO	Left	-40	-30	12	9.462	
Occipital cortex	Calcarine, LG	Right	18	-98	-4	9.949	1134
	MOG	Right	28	-92	0	9.614	
	IOG	Right	36	-82	-10	7.638	
Occipital cortex	IOG, calcarine gyrus,	Left	-22	-94	-2	9.806	788
STG	Supramarginal gyrus, MTG	Right	64	-28	10	9.547	1263
	Heschl’s gyrus, RO	Right	52	-20	8	8.750	
SMA	MCC	Left	-6	-4	54	8.981	692
	SMA, SFG	Right	8	6	56	6.262	
	SMA, SFG	Right	10	2	64	5.712	
Cerebellum *(69*.*6% in VI; 27*.*9% in V)*	V 56%; FG	Right	16	-50	-20	7.222	369
	VI 84%, FG	Right	28	-44	-28	6.183	
	VI 97%, FG	Right	28	-52	-24	6.084	
IFG	IFG pars Tri./pars oper.	Right	42	8	28	7.105	236
Cerebellum *(23*.*1% in VIIIb; 13*.*3% in VIIIa)*	VIIIa (Hem.) 33%, VIIIb (Hem) 24%; 23%	Right	22	-60	-48	6.936	79
	VIIIa (Hem.) 60%	Right	30	-54	-50	4.997	
Insula	IFG pars Tri./pars oper.	Right	34	22	8	5.621	25
Thalamus	Pallidum	Left	-10	-18	4	5.439	89
MCC	PCC		0	-40	36	5.209	22

Coordinates are listed in MNI space. Significance level: uncorrected *p* < .05 FWE corrected, cluster with at least 5 voxels. FG, fusiform gyrus; IFG: Inferior frontal gyrus; IOG: Inferior occipital gyrus; IPL: Inferior parietal lobule; LG, lingual gyrus; MCC: Middle cingulate cortex; MOG: middle occipital gyrus; MTG, middle temporal gyrus; PCC: Posterior cingulate cortex; PCG: postcentral gyrus; PRG: precentral gyrus; RO: rolandic operculum; SFG: Superior frontal gyrus; SMA: Supplementary motor area.

## Results

### Behavioral results

**Fig 2:**
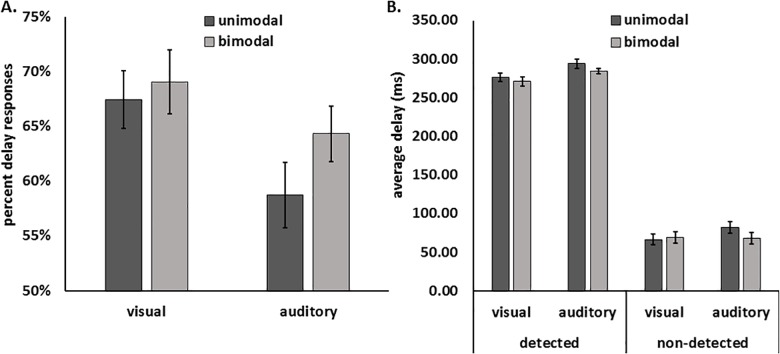
**Behavioural results as percent delay responses (A, left) and averaged delay per condition (B, right) across all participants.** In both tasks, bimodal trials showed more delay responses than unimodal trials. Furthermore, there was a trend for lower average delay for detected bimodal compared to detected unimodal trials, indicating that more trials with small delays had been detected in bimodal compared to unimodal conditions.

Fig 2 depicts behavioral performance as percent delay responses (A, left) and averaged delay per condition (B, right) across all participants. A repeated-measures ANOVA performed on the percent delay responses using the factors modality (unimodal vs bimodal) and task (visual vs auditory) revealed a significant main effect of modality and of task (F(1,19) = 6.809, p = 0.017, *η*^*2*^_*p*_ = 0.264 and F(1,19) = 9.541, p = 0.006, *η*^*2*^_*p*_ = 0.334, respectively). The interaction between these factors was not significant (F(1,19) = 2.861, p = 0.107, *η*^*2*^_*p*_ = 0.131). Analysis of the average delays per condition revealed significant main effects for detection (detected vs. undetected, F(1,19) = 1444.512, p < 0.001, *η*^*2*^_*p*_ = 0.987), task (auditory vs. visual, F(1,19) = 7.300, p = 0.014, *η*^*2*^_*p*_ = 0.278) and a trend for modality (unimodal vs. bimodal, F(1,19) = 4.274, p = 0.053, *η*^*2*^_*p*_ = 0.184). Additionally we revealed significant modality*task (F(1,19) = 4.455, p = 0.048, *η*^*2*^_*p*_ = 0.190) as well as detection*task interactions (F(1,19) = 4.597, p = 0.045, *η*^*2*^_*p*_ = 0.195). However, the modality*detection and modality*task*detection interaction did not reach significance (F(1,19) = 0.201, p = 0.659, *η*^*2*^_*p*_ = 0.010 and F(1,19) = 2.397, p = 0.138, *η*^*2*^_*p*_ = 0.112, respectively).

Analysis of ‘button press latencies’ (time used to press the button) per condition revealed no significant main effects (unimodal vs. bimodal, F(1,19) = 0.542, p = 0.470, *η*^*2*^_*p*_ = 0.028, and auditory vs. visual, F(1,19) = 3.903, p = 0.063, *η*^*2*^_*p*_ = 0.170) or modality*task interaction (F(1,19) = 0.353, p = 0.559, *η*^*2*^_*p*_ = 0.018). Furthermore, explorative correlation analyses revealed no significant correlation between the individual time used to press the button and performance in any condition.

Performance for the bimodal detection task during passive conditions was very high (PV: mean = 98.33%, SD = 4.36; PA: mean = 98.75%, SD = 3.05; PB: mean = 97.91%, SD = 3.70) and there were no significant differences between conditions (*p* > 0.494).

### fMRI results: Processing of action consequences compared to unpredictable control stimuli

**Fig 3:**
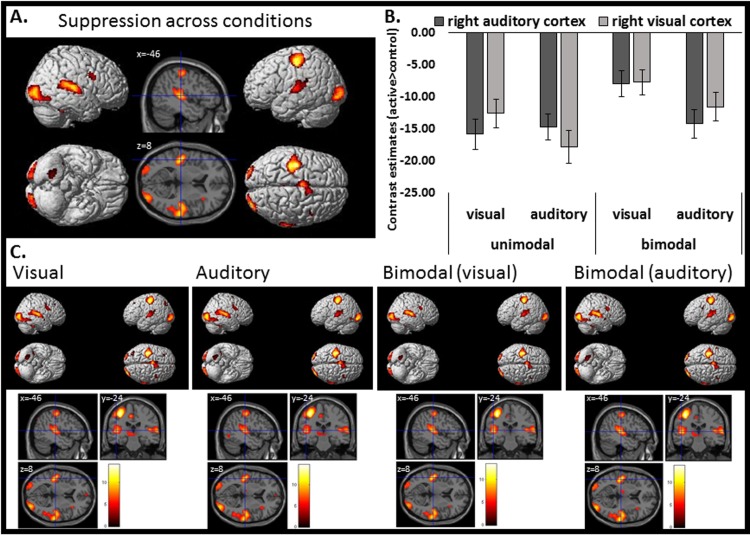
BOLD suppression for the processing of action consequences in contrast to unpredictable identical control stimuli. (A). Conjunction analysis for the suppression effect (active<control) across conditions (VU<VC ∩ AU<AC ∩ VB<BC ∩ AB<BC). (B). Contrast estimates (extracted eigenvariates) of the activation clusters in the right auditory (dark gray) and visual (light gray) cortex, respectively. Each bar represents the amount of suppression as contrast between active auditory, visual or audio-visual consequences minus respective auditory, visual or audio-visual control conditions. (C). Suppression effects for each individual condition. VU: visual unimodal, AU: auditory unimodal, VB: visual bimodal, AB: auditory bimodal, VC: visual unimodal control, AC: auditory unimodal control, BC: bimodal control. P < .05, FWE corrected for multiple comparisons. Error bars represent the standard error of the mean.

The comparison of the responses to action consequences (active conditions) compared to the responses to unpredictable control stimuli (passive conditions) revealed for each condition (see Fig 3B and 3C; VU<VC; AU<AC; VB<BC; AB<BC) activation reduction in the active conditions in a widespread neural network, including bilateral posterior occipital cortices, bilateral temporal cortices and predominantly left motor cortical areas. Conjunction analyses across conditions (VU<VC ∩ AU<AC ∩ VB<BC ∩ AB<BC) suggest that this suppression effect is quite independent of task or stimulus modality (see Table 1, Fig 3A). The inverse contrast (active>control) revealed activity in the right pre-/postcentral gyrus (MNI: x = 38, y = -22, z = 54; t = 9.72; cluster extension = 796 voxels), the left medial occipital lobe (MNI: x = -4, y = -86, z = -8; t = 7.91; cluster extension = 2742 voxels), lingual gyrus/precuneus (MNI: x = 12, y = -54, z = 2; t = 5.29; cluster extension = 36 voxels) and the left hippocampus (MNI: x = -26, y = -36, z = 10; t = 5.13; cluster extension = 8 voxels).

When button presses were not included as condition of no interest the contrast active>control revealed predominantly broad activation of the left motor cortex (MNI: x = -44, y = -18, z = 64; t = 10.45; cluster extension = 6336 voxels), reflecting the right hand finger movement. The general suppression effect (control>active) in the bilateral visual (MNI: x = 38, y = -54, z = -14; t = 5.21, p = 0.012 FWE, MNI: x = 46, y = -52, z = -12; t = 4.32, p = 0.258 FWE) and auditory cortices (MNI: x = 52, y = -50, z = 18; t = 4.69, p = 0.080 FWE; MNI: x = -64, y = -44, z = 10; t = 2.70, p = 0.004 uncorrected) was weaker, but still present. However, the predominantly left motor cortical suppression effect switched to the right hemisphere (MNI: x = 36, y = -16, z = 52; t = 5.46; cluster extension = 860 voxels). Thus, activation of the active/passive comparison, especially in the motor cortices, has to be interpreted with caution.

#### Correlation of activation suppression and behavioral data

Regarding delay responses, we found for the VB condition a negative relationship between proportion of delay responses and activation in the left (r = -0.507, p = 0.023, two tailed, uncorrected) and right visual cortex (r = -0.534, p = 0.015, two tailed, uncorrected). For the VU condition only the negative relationship between proportion of delay responses and activation in the right visual cortex was significant (r = -0.467, p = 0.038, two tailed, uncorrected; see Table A in S1 File for all results). This result indicates that lower neural activation (stronger suppression) is related to better performance (increased proportion of detected delays), speaking for a more efficient processing (at least in the visual task conditions). No significant positive correlations were observed. Thus, it is unlikely that activation reduction in active conditions reflects simply an interference with (or distraction due to) the additional button press task.

Corresponding to the correlations with the proportion of delay responses, we found for the VB condition a positive relationship between the average delays for detected trial and activation in the left (r = 0.693, p < 0.001, two tailed, uncorrected) and right visual cortex (r = -0.689, p < 0.001, two tailed, uncorrected). For the VU condition the positive relationship between average delay and activation in the right visual cortex reached significance at a trend level (r = 0.385, p = 0.094, two tailed, uncorrected; see Table B in S1 File for all results). This result indicates that lower neural activation (stronger suppression) is related to better performance (reduction of average delays), as shorter delays were detected. Interestingly activation in auditory cortices was positively correlated to average delays in the VB condition, too (left r = 0.487, p = 0.029; right r = 0.526, p = 0.017). No significant negative correlations were observed.

### fMRI results: Processing of subjective delayed (delay detected) and undelayed (delay undetected) trials.

**Fig 4:**
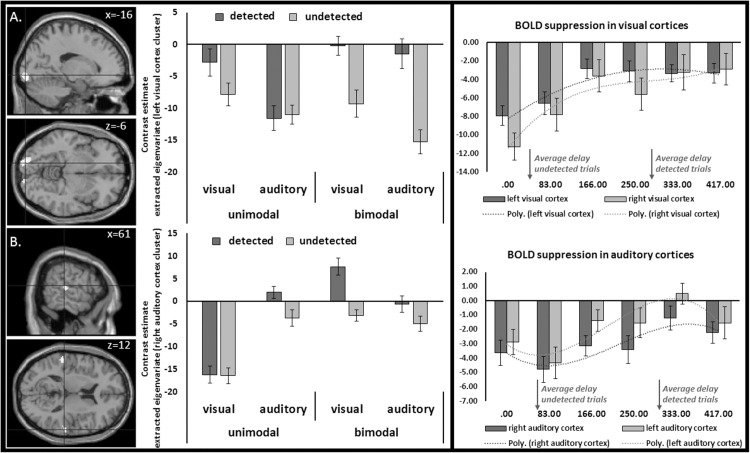
**Suppression effects for subjectively undelayed (undetected delay) compared to delayed (detected delay) trials in visual (A, top) and auditory (B, bottom) cortices.** Data are inclusively masked by the suppression effect illustrated in Fig 3A. The lack of effects in the visual cortex for auditory unimodal trials and in the auditory cortex for visual unimodal trials may be due to the fact that detected trials for these conditions led to high activation in brain regions related to the respective task modality only. Interestingly, in bimodal trials suppression was observed in both modalities. Bar graphs at the right illustrate suppression effects in visual (top) and auditory (bottom) cortices across conditions as a function of the delay between the action and the stimulus. Error bars represent the standard error of the mean. P < 0.001 uncorrected with a cluster extend of 50 voxels.

In the second analysis, for each active condition, trials where delays had been detected (VU-d, AU-d, VB-d, AB-d) were separated from trials where delays had not been detected (VU-nd, AU-nd, VB-nd, AB-nd). With this analysis we first tested more specifically for BOLD suppression for undetected conditions (detected > undetected, masked) in primary sensory brain regions by applying an inclusive masking procedure using the result pattern of the first analyses (conjunction analyses; see Fig 3A) as mask. We found no effects by applying the conservative FWE correction for multiple comparisons. However, on the more liberal threshold (p < 0.001 uncorrected, 50 voxels) we found indeed BOLD suppression (detected>undetected) in bilateral occipital (MNI: x = -16, y = -96, z = -6; t = 4.76, cluster extension = 311 voxels, p < .012 FWE cluster correction; MNI: x = 24, y = -92, z = -14; t = 4.31; cluster extension = 99 voxels) and temporal (MNI: x = 60, y = -28, z = 8; t = 4.37; cluster extension = 81 voxels; MNI: x = -56, y = -32, z = 12; t = 3.88; cluster extension = 60 voxels) brain regions (see Fig 4). These data are in line with the hypothesis that the better prediction for undelayed trials (e.g., due to the temporal contiguity) lead to greater activation reduction in auditory and visual cortices compared to more unpredictable delayed trials. Note, this analyses was less affected by the button press condition of no interest. We found also BOLD suppression (detected>undetected) in bilateral occipital (MNI: x = -16, y = -94, z = -6; t = 4.22; MNI: x = 26, y = -92, z = -12; t = 3.59) and temporal (MNI: x = 60, y = -28, z = 8; t = 3.65; MNI: x = -52, y = -32, z = 14; t = 3.08) brain regions when not controlling for the button press.

**Fig 5:**
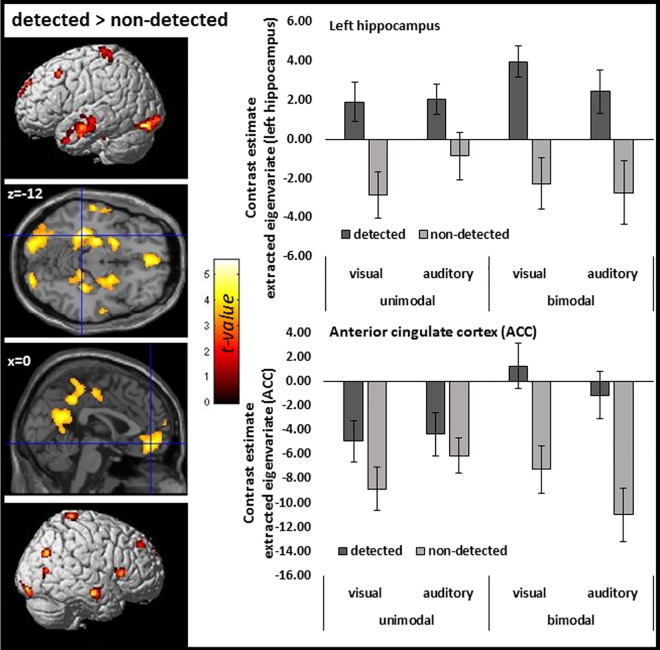
FMRI results for subjectively undelayed (undetected delay) compared to delayed (detected delay) trials (p < 0.001 uncorrected with a cluster extent of 50 voxels). The bar graph illustrates the contrast estimates of the left hippocampus cluster (at p < 0.05 FWE) and the bilateral ACC cluster (p < 0.001 uncorrected; for statistics see Table 2).

For general neural processes related to the detection of delays (detected > undetected; unmasked), we found effects in the left parahippocampus (MNI: x = -30, y = -34, z = -12; t = 5.58; cluster extension = 27 voxels), the right precuneus (MNI: x = 14, y = -60, z = 22; t = 5.53; cluster extension = 19 voxels) and the left putamen/insula (MNI: x = -28, y = -2, z = -2; t = 5.16; cluster extension = 7 voxels). At a more liberal threshold (p < 0.001, 50 voxels), we revealed a more distributed network comprising the medial prefrontal lobe and the anterior and posterior cingulate cortex (ACC/PCC), the temporal poles, as well as parietal and hippocampal structures (see Fig 5 and Table 2). The opposite contrast (undetected>detected) revealed two clusters of activation in the right inferior frontal gyrus (MNI: x = 52, y = 20, z = 6; t = 4.42, p < 0.001 uncorrected; cluster extension = 149 voxels; MNI: x = 34, y = 26, z = -6; t = 3.93, p < 0.001 uncorrected; cluster extension = 73 voxels).

**Table 2:** Processing of subjective delayed (delay detected) and undelayed (delay undetected) trials at a liberal threshold (*p* < .001 uncorrected, cluster extent: 50 voxels).

Anatomical Region			Coordinates				no. Voxels	*P* peak
	Cluster extent	Hem.	*x*	*y*	*z*	*t*-value		FWE
PH	HC	Left	-30	-34	-12	5.580	1925	0.003
	Putamen, Pallidum, Insula	Left	-28	-2	-2	5.160		0.017
	HC, Amygdala	Left	-22	-16	-12	4.720		0.083
Precuneus		Right	14	-60	22	5.530	3807	0.004
	Precuneus	Left	-4	-62	18	4.790		0.065
	Calcarine gyrus	Left	-6	-56	4	4.690		0.092
MTG	ITG, TP	Right	64	-6	-20	5.160	170	0.017
	MTG, STG	Right	60	-12	-16	4.610		0.119
MTG	STG, ITG	Left	-60	-8	-18	4.930	616	0.039
	MTG, STG, ITG	Left	-64	-20	-8	4.540		0.151
	TP	Left	-46	10	-34	4.320		0.293
Calcarine gyrus	IOG, MOG	Left	-16	-96	-6	4.760	766	0.073
	LG	Left	-24	-86	-14	4.280		0.328
	FG	Left	-32	-80	-14	3.760		0.855
MFG	SFG, MFG	Right	32	44	42	4.620	80	0.117
	MFG, Rectus		0	48	-12	4.490	1005	0.176
	MFG	Left	-4	58	-4	4.270		0.336
	ACC	Left	-4	38	-6	3.930		0.694
IOG	LG, FG	Right	30	-90	-16	4.480	183	0.184
	Calcarine gyrus, LG	Right	12	-98	-6	3.680		0.910
Angular gyrus	MOG, IPL	Right	50	-68	32	4.460	178	0.195
Putamen	Pallidum, Amygdala	Right	28	0	-6	4.420	1034	0.219
	HC, ParaHC, FG	Right	34	-30	-10	4.380		0.247
	Putamen, Insula, pallidum	Right	34	-6	-6	4.210		0.392
STG	MTG, RO	Right	60	-28	8	4.370	81	0.254
	STG, Heschl’s gyrus, RO	Right	54	-22	6	3.270		0.999
TP	ITG	Right	48	16	-32	4.290	54	0.320
	ITG, MTG, TP	Right	52	4	-36	3.220		1.000
MFG	Precentral gyrus, IFG,	Left	-38	20	46	4.230	116	0.368
SFG	Medial SFG, MFG	Left	-12	58	36	4.160	189	0.440
	mSFG, SFG	Left	-10	66	24	3.800		0.825
	mSFG	Left	-8	48	50	3.460		0.987
LG	Cerebellum, PH	Right	18	-36	-12	4.020	209	0.592
	LG, FG, PH	Right	24	-50	-8	3.800		0.819
	Vermis, LG	Right	2	-30	-10	3.190		1.000
ITG	FG, Cerebellum	Right	44	-48	-24	4.000	53	0.620
PCG	PRG, SPL	Left	-28	-32	76	3.970	462	0.646
	PCG, SPL, Precuneus	Left	-24	-38	66	3.840		0.783
	Paracentral gyrus, PCG, PRG	Left	-12	-30	82	3.770		0.843
STG	MTG, SMA	Left	-56	-32	12	3.880	71	0.745
MTG	STG, ITG	Right	48	-70	8	3.860	120	0.765
	MOG, MTG, IOG	Right	40	-72	0	3.400		0.994
MTG	STG, RO	Left	-46	-40	6	3.660	83	0.920
	MTG, RO, Heschl’s gyrus	Left	-40	-36	16	3.650		0.928
	MTG, STG, ITG	Left	-48	-48	6	3.470		0.986
MOG	Angular gyrus, IPL	Left	-44	-74	34	3.450	68	0.989
	Angular gyrus, MOG, MTG	Left	-44	-66	24	3.400		0.994
	Angular gyrus, MOG, IPL	Left	-40	-70	40	3.210		1.000

Coordinates are listed in MNI space. ACC: Anterior cingulate cortex; FG, fusiform gyrus; IFG: Inferior frontal gyrus; IPL: Inferior parietal lobule; IOG: Inferior occipital gyrus; ITG, inferior temporal gyrus; LG, lingual gyrus; MOL: Medial occipital lobe; MOG: middle occipital gyrus; MFG: Medial frontal gyrus; MTG, middle temporal gyrus; PCG: postcentral gyrus; PG, parahippocampal gyrus; PRG: precentral gyrus; RO: rolandic operculum; SFG: Superior frontal gyrus; SOG: superior occipital gyrus; SPL: Superior parietal lobule; STG, superior temporal gyrus; TP: Temporal pole.

Control analyses comparing detected versus undetected trials matched for delay revealed a similar pattern of activation as illustrated in Figs 4 and 5 (detected>undetected; see Fig B in S1 File). Although only the 167ms delay could be included in this post-hoc control analysis, these results suggest that the previously reported results are not just due to the physical delay, but are also related to awareness of delay.

#### fMRI results: Interaction effects

**Fig 6:**
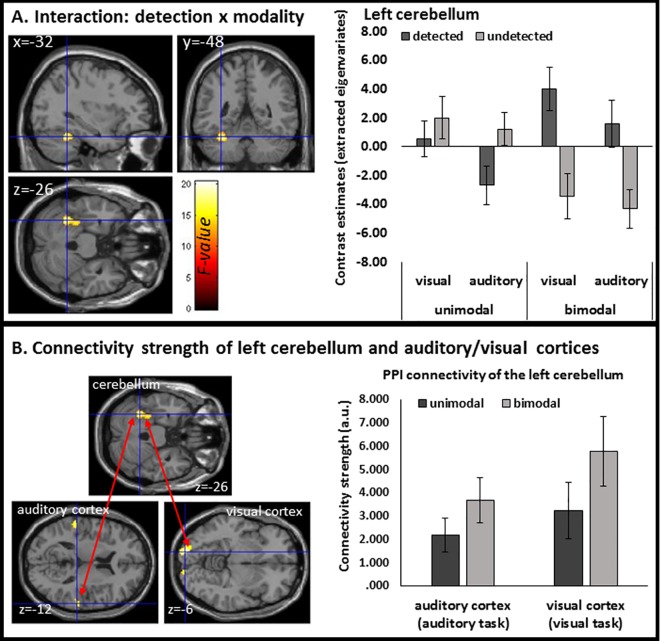
The cerebellum: Interaction and PPI results. (A) Activation of the left cerebellum with cluster extensions in the left fusiform gyrus for the interaction of delay detection (detected/undetected) and modality (unimodal/bimodal). Contrast estimates (extracted eigenvariates) of the respective cluster (bar graph on the left) illustrate a specific activation for detected compared to undetected trials in the bimodal conditions (independent of task modality). Error bars represent the standard error of the mean. P < 0.001 uncorrected with a cluster extent of 50 voxels. (B) Connectivity results (PPI analyses) for the left cerebellum and seed regions in the right auditory (Fig 4B) and left visual cortex (Fig 4A). The bar graph illustrates the connectivity strength (arbitrary units, a.u.) of the cerebellum cluster (extracted eigenvariates from the PPI group analyses) and respective seed regions for unimodal (dark gray) and bimodal (light gray) conditions. Connectivity strength increased in bimodal conditions probably due to the additional task irrelevant stimulus.

Interaction analyses were applied to explore the effect of task (visual/auditory) and modality (unimodal/bimodal) on the neural processing of trials subjectively perceived as delayed compared to undelayed (detected/undetected). We found no effects by applying the conservative FWE correction for multiple comparisons. However, at a more liberal threshold (p < 0.001 uncorrected, 50 voxels) we found a significant interaction effect for task (auditory/visual) by modality (unimodal/bimodal) in the left cerebellum (62.0% in left lobule VI (Hem.), 9.3% in lobule V (Hem.)) with cluster extensions to the fusiform gyrus (16.7% in area FG3, MNI: x = -32, y = -48, z = -26; F = 20.41, cluster extension = 182 voxels, p < 0.053 FWE cluster corrected; see Fig 6). Contrast estimates of the respective cluster (extracted eigenvariates; bar graph on the left in Fig 6) illustrate a specific activation for detected compared to undetected trials in the bimodal conditions (independent of task modality). This effect is mainly driven by significant differences between detected and undetected trials in the bimodal conditions (detected>undetected, MNI: x = -30, y = -34, z = -28; t = 5.82, cluster extension = 156 voxels, p < 0.001 FWE corrected) and no significant modulation in the unimodal conditions (p > 0.001 uncorrected). No other interaction effect revealed significant results at the chosen threshold (p < 0.001 uncorrected, 50 voxels).

To further understand how the neural processing of auditory and visual action outcomes is related to the neural processing in the left cerebellum, we additionally conducted exploratory connectivity analyses in the form of psychophysiological interaction (PPI) analyses. Seed regions in the right auditory (Fig 4B) and left visual cortex (Fig 4A) were selected, as they demonstrated the most prominent suppression effect (highest t-values in the second analyses, see above) in the auditory and visual cortices (see Fig 4). To test for specific effects of bimodal vs. unimodal conditions on connectivity strength between the seed regions and the left cerebellum, eigenvariates of the left cerebellum cluster (identified in the stimulus type*detected interaction; see Fig 6A) were extracted from respective PPI analyses and further analyzed using SPSS. A repeated-measures ANOVA performed on the extracted data using the factors modality (unimodal vs. bimodal) and audio/visual processing (visual vs. auditory cortex) revealed a significant main effect of modality (F(1,19) = 5.411, p = 0.031, *η*^*2*^_*p*_ = 0.222), indicating increased connectivity in bimodal compared to unimodal conditions (see Fig 6B). The main effect audio/visual processing (F(1,19) = 1.677, p = 0.211, *η*^*2*^_*p*_ = 0.081) and the interaction between these factors were not significant (F(1,19) = 0.259, p = 0.617, *η*^*2*^_*p*_ = 0.013). Connectivity strength increased in bimodal conditions (see Fig 6B) probably due to the additional task-irrelevant stimulus.

Whereas exploratory analyses for the left motor cortex (pre-/postcentral gyrus, PRG/PCG) and SMA (see Fig 3A, Table 1 cluster 1 and 6) revealed general positive connectivity to the seed regions in the auditory and visual cortices (for all conditions: one sample t-tests, p < .05 uncorrected), no significant main effects (PRG/PCG audio/visual processing: F(1,19) = 0.010, p = 0.922, *η*^*2*^ = 0.001; PRG/PCG unimodal/bimodal: F(1,19) = 1.542, p = 0.229, *η*^*2*^ = 0.075; SMA audio/visual processing: F(1,19) = 2.488, p = 0.131, *η*^*2*^ = 0.116; SMA unimodal/bimodal: F(1,19) = 0.346, p = 0.563, *η*^*2*^ = 0.018) or interactions were found regarding task or modality (PRG/PCG: F(1,19) = 0.859, p = 0.366, *η*^*2*^ = 0.043; SMA: F(1,19) = 0.078, p = 0.783, *η*^*2*^ = 0.004).

## Discussion

Performing an action and processing its consequences are usually tightly coupled, making those consequences more predictable than other external events. However, whether and how we predict multisensory action outcomes remains largely unknown. To shed light on this issue, we investigated the neural processing of multisensory consequences of one’s own action using unimodal and bimodal visual and auditory stimuli presented at various delays after a button press, and identical, but action unrelated, unpredictable control stimuli. We observed BOLD suppression in a broad network including bilateral auditory, visual, and sensorimotor brain regions for action consequences compared to the responses to identical, but unpredictable, control stimuli. Suppression was independent of task or stimulus modality and was strongest for subjectively undelayed stimuli. An interaction of modality (unimodal vs. bimodal) by delay detection (detected vs. undetected) revealed activation in the left cerebellum with cluster extensions in the fusiform gyrus. Thus, the internal model and related cerebellar functions prepare the perceptual system for all possible action consequences and probably underlie the behavioral advantage for bimodal versus unimodal conditions.

### Cross-modal action-related suppression

Previous studies showing action-related suppression (or corresponding increase of activation for delayed feedback) in the auditory, visual, and somatosensory system have tested these modalities separately (e.g., [10, 11, 14, 16, 17, 32, 40]). On the other hand cross-modal audio-visual suppression effects have been reported, but independent of action [49]. Our data extend these previous results in demonstrating action related BOLD suppression for more than one modality (in auditory and visual cortices) at the same time. In our paradigm, auditory and visual action consequences were equally likely. Consequently, visual and auditory information were equally predictable following a self-initiated button press.

It has been suggested that the efference copy plays an important role in predicting the sensory consequences of actions, such as various hand movements [14, 50–52]. Many studies have focused on the role of this forward model in predicting visual [14, 50–52], tactile [25, 32], and auditory [53, 54] consequences. We found BOLD suppression in both auditory and visual areas after either or both auditory and visual stimuli related to active movement, which suggests that the sensory system is prepared to process any sensory information consequent to a button press. Exploratory correlation analyses suggest that lower neural activation (stronger suppression) in visual cortices was related to better performance (higher detection rate and reduced average delay in detected trial) predominantly for the bimodal visual task condition, speaking for a more efficient processing. Individual differences in multisensory integration and especially the temporal aspects of multisensory binding received increasing attention in recent years, suggesting practical and clinical relevance [55]. It has been shown, that variations in the temporal binding window (limited range of asynchronies tolerated for perceptual binding) are related to an individual’s ability to integrate multisensory cues [56]. Our data suggest a relationship between individual differences in temporal processing of action outcomes and BOLD suppression in sensory cortices. Thus, the association of action related predictive mechanisms and individual differences in temporal and multisensory processing remains an important topic for future studies.

No previous studies have directly tested the prediction of multisensory consequences of one’s own action at the neural level. However, a previous behavioural study from our group found that bimodal action consequences led to an enhancement in the detection of delays between action and feedback, compared to unimodal action consequences, in particular when the task irrelevant stimulus was presented close to the action [24]. This was interpreted as evidence that the forward model creates predictions for multiple modalities. Here we could replicate the behavioural finding (bimodal enhancement) and extend it to new evidence about the neural correlates. Another behavioural study showed that unpredicted visual stimuli affected loudness perception of auditory stimuli, both for self-generated stimuli and stimuli predicted by a cue [57]. However, this study investigated the general cross-modal effect of predictability of task-irrelevant stimuli on the perception of the task stimuli without using fMRI methods. In our study, we were specifically interested in the perception of multisensory action consequences compared to unpredictable control stimuli. Few other behavioral studies have included multisensory action consequences to study the sense of agency. For example, Farrer and colleagues found that the presentation of a sound at the time of the button press significantly reduced the thresholds at which participants felt in full control of the appearance of the visual stimulus [58]. Similarly, lower thresholds were found when additional tones were presented at the time of the button press and visual stimulus in a cross-modal grouping paradigm with variable delayed visual stimuli [59]. In line with previous behavioural data [24] our findings point towards the idea that one forward model creates multisensory predictions which consequently leads to bimodal facilitation on a behavioural level and activation reduction in both auditory and visual cortices.

### The temporal window of suppression

Trials in which the participant perceived stimuli temporally aligned with their action (undetected) were accompanied by less neural responses in sensory brain areas as the stimuli that subjects perceived as presented with a delay after their button press. Thus, we observed more BOLD suppression in sensory brain areas when action consequences occurred close to action and were perceived as undelayed. As the task was to detect any delay in sensory feedback, this contrast reflects activity for detected violation of temporal contiguity between action and feedback. Framed differently, the violation of temporal prediction led to activation increase in brain regions relevant for the processing of auditory and visual information. By comparing detected and non-detected trials we could connect BOLD suppression more directly to action, since timing between action and its sensory consequence matters. Suppression was strongest in highly predictable trials in which the participants could detect no delay between action and feedback. That timing matters for sensory suppression could also be demonstrated for example by a MEG study, where N100m suppression in response to pure tones was especially pronounced immediately after articulary lip movements [60]. This finding has been interpreted as suppression in the auditory cortex being caused by an efference copy from the speech-production system, generated during both own speech and lipreading [60]. Increased BOLD activity when feedback was delayed and/or the delay was detected has been observed in visual [11, 14, 16], auditory [17], and tactile [32] modalities. However, to our knowledge, the present study is the first to demonstrate this effect for bimodal audio-visual conditions too.

### The neural basis of cognitive factors

The broad network in which we found differences between detected and non-detected trials included the bilateral hippocampus, the anterior and posterior cingulate cortices (ACC, PCC), parietal structures, and the temporal poles. It has been suggested that sensory attenuation is reflected in modulation of both sensory processing (e.g., for auditory or visual stimuli) and processing associated with a reduced engagement of cognitive control in response to an expected sensory event [61]. This latter modulation could thus be seen as neural processing associated with predictability, such that it is attenuated for predicted stimuli but might also be increased for unexpected stimuli. Thus, frontal, parietal and hippocampal activations for detected compared to non-detected delay trials might reflect cognitive control processes. However, the observed activation pattern including midline structure activations (ACC/PCC) also corresponds to the so-called ‘self-referential network’ [62, 63]. Thus, self-referential processing load might be especially high when consequences of our own actions deviate from our temporal prediction. Since our participants were explicitly told that they were always the agent, they would have attributed even delayed feedback as the audio/visual consequences of self-action but this would have been in conflict with the usual expectation of zero delay. ACC activation has been found to be involved in conflict monitoring [64] and its activation here could therefore be a consequence of a prediction of error [65]. Thus, activation for trials where delays were detected versus trials where delays were not detected could either reflect conflict monitoring, cognitive control processes in response to an unexpected sensory event, or a high self-referential processing load.

### The role of the cerebellum

In addition to the main effect ‘delay detection’ discussed above, we found a significant interaction of delay detection (detected/non-detected) and modality (unimodal/bimodal) in activation of the left cerebellum (VII) with cluster extensions in the left fusiform gyrus. Contrast estimates of the respective cluster (see bar graph Fig 6) illustrate a specific activation for detected compared to non-detected trials in the bimodal conditions (independent of task modality), an effect that was absent in the unimodal condition. Notably the right cerebellum (VI and VIII) seems to be generally involved across conditions (see Table 1 and Table 2), however, the left cerebellum (VII) seems to be specifically involved in predicting multisensory consequences of one’s own actions. The role of the cerebellum for action feedback prediction has been suggested [66] and supported by a number of imaging studies focusing on visual [14, 16] and tactile modalities [9, 10, 32]. We extend these findings by demonstrating for the first time a specific effect in the left cerebellum related to the processing of multisensory information produced by one’s own actions. The observed activation pattern in the cerebellum could also reflect a multisensory comparator mechanism as it compares expected and perceived auditory-visual signals (e.g., [32]). It has been proposed that the cerebellum is an important component of the system that provides precise predictions of the sensory consequences of motor commands and acts as a comparator between intended and achieved movement, signalling errors in motor performance and neurophysiological data [32, 67]. In contrast to previous investigations we provide evidence for a specific role of the left cerebellum in processing multisensory action outcomes. Moreover, this effect was not only absent in the unimodal conditions, but also independent of task modality; i.e. we revealed more activation for detected compared to non-detected delay trials in the cerebellum for both auditory and visual task conditions. Thus, the activation of the left cerebellum might be relevant for explaining the behavioural differences between unimodal and bimodal conditions. Behaviourally, we observed an advantage for bimodal trials, as shown by a significant increase in detection rates compared to unimodal conditions. These behavioural results are in line with our recent behavioural study [24] and suggest that the forward model generates predictions for auditory AND visual modalities, leading to an advantage for delay detection in bimodal trials. This bimodal advantage might be due to a specific multisensory predictive function of the cerebellum.

In line with our data, cerebellar activity during tasks involving crossmodal matching had been reported [23, 68–70]. For example, it has been observed that combined audiovisual motion detection led to increased activity bilaterally in cerebellar lobule VI and right lateral crus I, relative to unimodal visual and auditory motion tasks [68]. In an earlier study, subjects’ ability to detect crossmodal temporal mismatch between simple stationary auditory and visual stimuli was assessed in two separate auditory–visual (AV) and visual–auditory (VA) conditions. Brain regions activated in common to both (AV and VA) conditions, included the left cerebellum [69]. Together, these results suggest that the cerebellar hemispheres play a role in the detection of multisensory invariant temporal features in concurrent streams of audio-visual information [23].

The PPI analysis suggests that the connectivity between activity of the sensory cortex, which was relevant for the processing of the target stimulus, and the left cerebellum increased in bimodal compared to unimodal conditions. Thus, the task irrelevant stimulus strengthens the functional connectivity (FC). Previous studies focussing on the FC of the cerebellum used resting-state activity (see [23] for an overview). These methods have contributed to distinguish two anatomic-functional parts of the cerebellum [71]: a sensorimotor region (lobules V–VI and VIII) and a multimodal cognitive and limbic region (lobule VIIA, especially crus I and II, with adjacent parts of lobule VI and VIIB, and lobule IX). In line with our result FC of the cerebellum to the visual [71–73] and auditory cortex [71, 72] had been found. A hypothesis is that the cerebellum aids information processing by making predictions, in the form of an “internal model” of sensory events [32, 74]. Alternatively it has been proposed that the cerebellum facilitates perception by monitoring and coordinating the acquisition of sensory information (see the section by Bower, in [23]). A third theory is that the cerebellum functions as an internal timing device for both motor and perceptual processes, with different areas of the cerebellum thought to provide separate timing computations for different tasks [75]. Whereas the differentiation of these theoretical accounts is beyond the scope of the current study, our findings support the relevance of the cerebellum for visual and auditory processing, timing, and specifically the prediction and processing of multisensory action consequences. Whereas activity in the left motor cortex and SMA are also related to auditory and visual cortices, no bi-modality specific effects (as for the cerebellum) could be observed. Thus, the cerebellum generates predictions specifically for multisensory action outcomes, reflected in its increased connectivity to task relevant sensory cortices and neural suppression for subjectively delayed compared to undelayed trials. Ultimately this predictive mechanism might lead to better delay detection rates in bimodal conditions.

### Limitations

Despite the new relevant findings and obvious advantages of our current approach it is important to mention some limitations. They include the relatively abstract stimulus material (button press, dot, and tone), and the fact that our design cannot distinguish between multisensory predictions due to efference copy mechanisms and multisensory predictions due to general temporal predictive mechanisms based on an intentional button press. A passive movement condition would be necessary to test more specifically for the role of efference copy. Such a condition is technically challenging to apply in an MRI environment; however, in a recent behavioral experiment, we did implement a passive movement condition which provides support for the involvement of efference copy in multisensory facilitation [24]. Within our present fMRI design, an alternative explanation for activation reduction in the active compared to the control conditions could simply be that the button press distracts from the perceptual task. Thus, less neural resources are left to process the auditory and visual stimuli. However, the exploratory correlation analyses demonstrate no positive relationship between BOLD suppression and delay detection rate as well as no negative relationship between BOLD suppression and the average delay of detected trials. For the visual conditions, better performance was correlated with reduced activation in visual sensory cortices suggesting a more efficient processing and arguing strongly against the distraction hypothesis. Nevertheless, the relationship between performance and suppression remains a relevant future research topic. Furthermore, the control of general button press effects is challenging in the applied design, due to the differences in active (button press) and control conditions (no button press) as well as the high temporal correlation between button press and auditory and visual feedback. Consequently, the fMRI analyses considering the button press compared to those neglecting its influence led to changes in the result pattern, predominantly in -but not restricted to- the motor cortices. A better balanced experimental designs and the use of a passive movement device might help to reduce these effects in future. Future studies should also extend our findings to natural outcomes and less constrained actions. However, in a world in which we are surrounded by computers and other devices, it is a common action to press a button and expect a visual and/or auditory consequence, such as when typing a letter or playing a game. Thus, despite the setup being fairly abstract, it can still be considered ecologically valid (c.f., [24]). Our study is an important first step in unravelling the neural processing of multisensory action consequences.

## Conclusions

In summary, our results support the existence of multisensory predictive mechanisms in a context where actions can have outcomes in different modalities. We observed BOLD suppression in auditory and visual sensory processing areas for action consequences compared to identical but unpredictable auditory/visual control stimuli and for trials perceived as simultaneous compared to trials in which delays had been detected. Thus, the internal model prepares the perceptual system for all possible action consequences and underlies the behavioural advantage for bimodal versus unimodal conditions. Our results suggest that the left cerebellum is especially relevant for the processing of violations in temporal contiguity between actions and its multisensory consequences. These new results highlight the relevance of multisensory predictive mechanisms for the understanding of how we act in and perceive the world.

## Supporting Information

S1 File Table A, Table B, Fig A, Fig B. (DOCX)
