# Supplementary material for: Predicting the Multisensory Consequences of One’s Own Action: BOLD Suppression in Auditory and Visual Cortices
Source: PLoS One. 2017 Jan 6;12(1):e0169131. doi: 10.1371/journal.pone.0169131 (PMC5218407; doi:10.1371/journal.pone.0169131)
Supplement: S1 File — (DOCX) [file pone.0169131.s001.docx]

**Supporting Information**

**S1 Table A.** *Correlations of BOLD suppression and behavioral performance (delay responses)*

|  |  | right visual cortex | left visual cortex | right auditory cortex | left auditory cortex |
| --- | --- | --- | --- | --- | --- |
| VU detection | r | **-.467*** | -.280 | ***-.407*** | -.335 |
|  | *sig. (2-tailed)* | **.038** | .232 | ***.075*** | .148 |
| VB detection | r | **-.534*** | **-.507*** | ***-.420*** | ***-.424*** |
|  | *sig. (2-tailed)* | **.015** | **.023** | ***.066*** | ***.062*** |
| AU detection | r | -.260 | -.196 | -.066 | -.133 |
|  | *sig. (2-tailed)* | .268 | .407 | .782 | .576 |
| AB detection | r | -.284 | -.229 | .033 | .073 |
|  | *sig. (2-tailed)* | .225 | .332 | .889 | .761 |

***S1 Table.*** *Spearman correlations (r) for individual differences in behavioral performance (delay responses) and average brain activation in auditory and visual cortices (extracted eigenvariates of the respective custers). VU: visual unimodal, AU: auditory unimodal, VB: visual bimodal, AB: auditory bimodal. We found only negative relationships between detection performance and activation in the left and right visual cortex. This result indicates lower neural activation (stronger suppression) is related to better performance (more delay responses), speaking for a more efficient processing. No significant positive correlations were observed. Thus, it is unlikely that activation reduction in active conditions reflects simply an interference with or distraction due to the additional button press task.*

**S1 Table B.** *Correlations of BOLD suppression and average delay for detected trials*

|  |  | right visual cortex | left visual cortex | right auditory cortex | left auditory cortex |
| --- | --- | --- | --- | --- | --- |
| VU av delay | r | ***.385*** | .168 | .302 | .186 |
|  | *sig. (2-tailed)* | ***.094*** | .478 | .195 | .431 |
| VB av delay | r | **.689***** | **.693***** | **.487*** | **.526*** |
|  | *sig. (2-tailed)* | **.001** | **.001** | **.029** | **.017** |
| AU av delay | r | .074 | .030 | .000 | -.027 |
|  | *sig. (2-tailed)* | .758 | .900 | 1.000 | .910 |
| AB av delay | r | .349 | .305 | .023 | -.062 |
|  | *sig. (2-tailed)* | .132 | .191 | .925 | .796 |

***S1 Table B.*** *Spearman correlations (r) for individual differences in behavioral performance (average delays) and average brain activation in auditory and visual cortices (extracted eigenvariates of the respective custers). VU: visual unimodal, AU: auditory unimodal, VB: visual bimodal, AB: auditory bimodal. We found only positive relationships between average delays and activation in the left and right visual cortex. This result indicates lower neural activation (stronger suppression) is related to better performance (lower average delays in detected trials), speaking for a more efficient processing. No significant negative correlations were observed. Thus, it is unlikely that activation reduction in active conditions reflects simply an interference with or distraction due to the additional button press task.*

**S1 Fig A:** *Results of the correlation analyses*


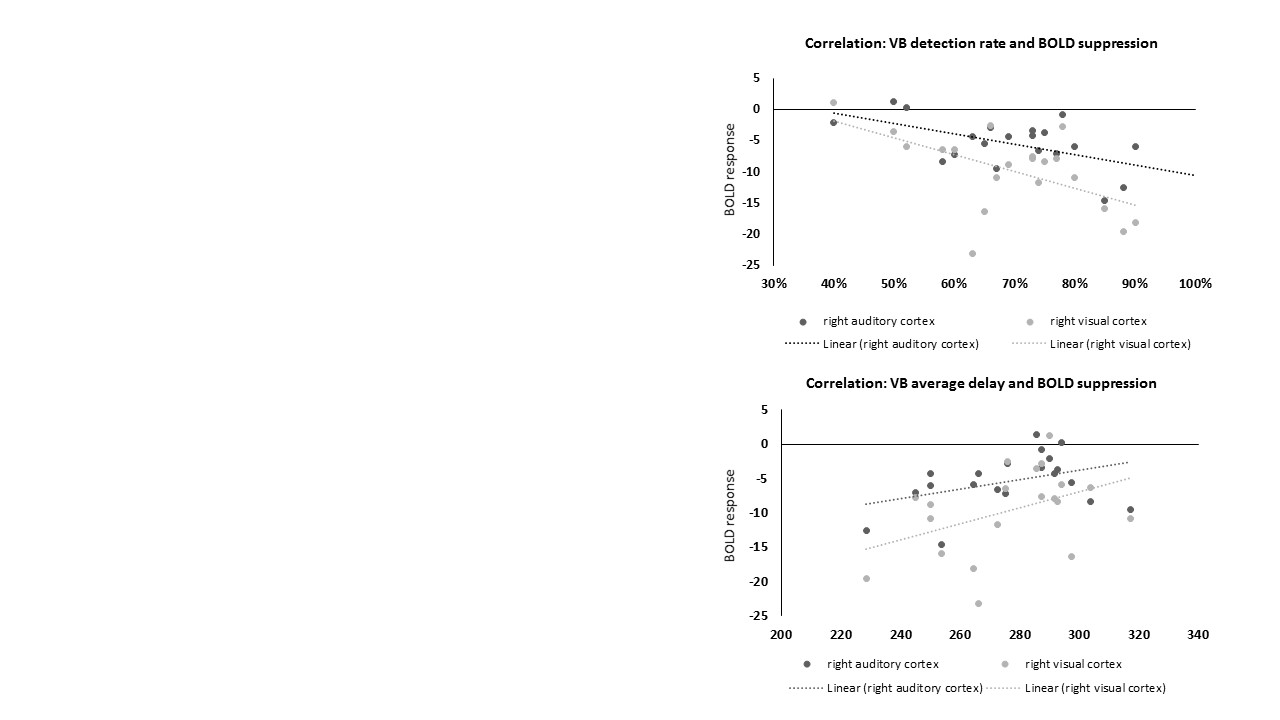


***S1 Fig A.*** *Illustration of the significant correlation of delay detection rate (top) and average delay (x axis, in milliseconds) for detected trials (bottom) of the bimodal condition during the visual task (VB) with BOLD responses (extracted eigenvariate of the respective cluster) in right auditory (dark gray) and visual (light gray) cortices.*

**S1 Fig B**: *FMRI results for subjectively undelayed (undetected delay) compared to delayed (detected delay) trials with a delay of 167 ms*


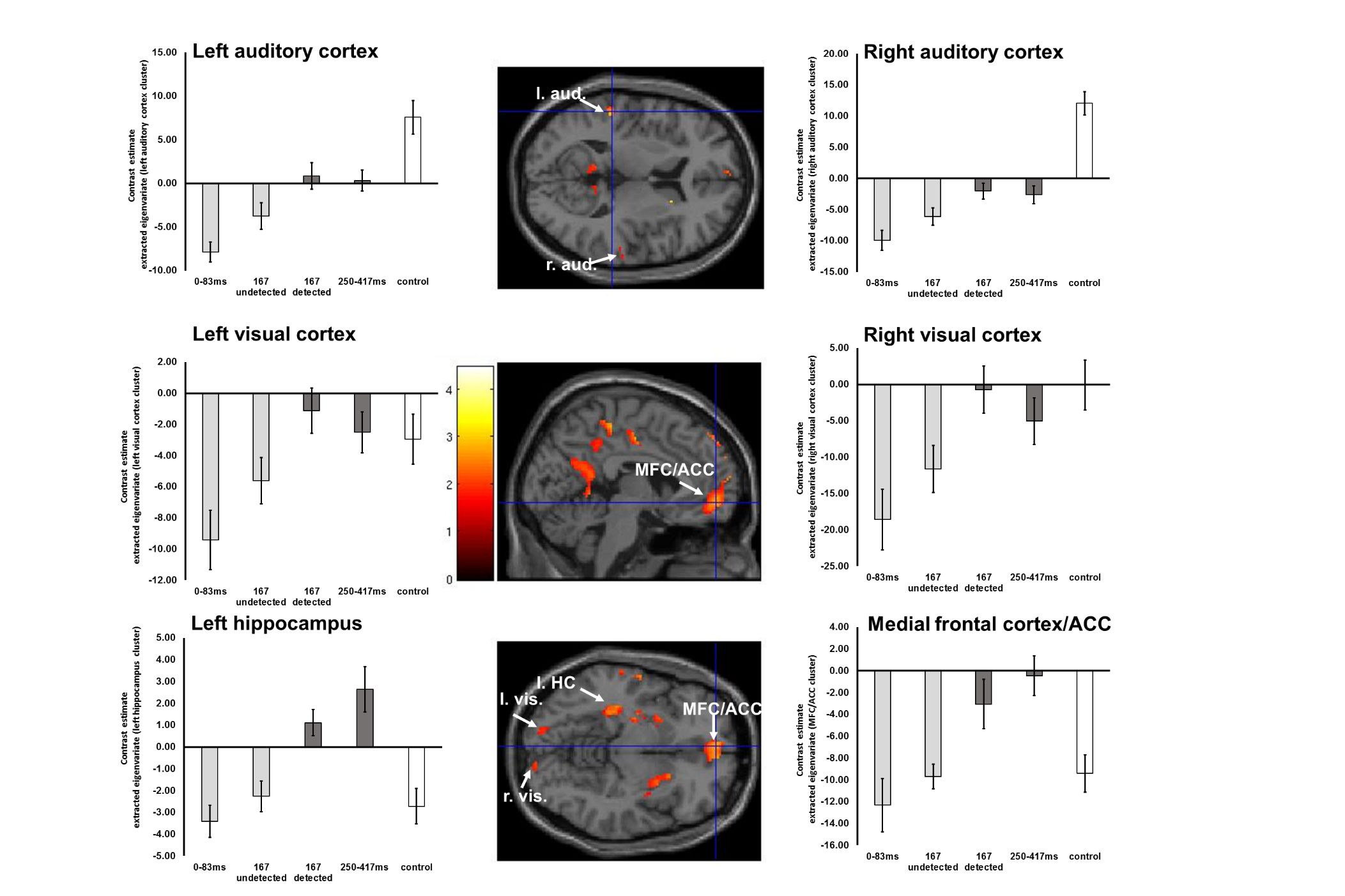


***S1 Fig B*** *illustrates the results of a control analyses comparing detected and undetected trials with the identical physical delay (167 ms) between action and stimulus. As in the main analysis (Fig. 4 and Fig. 5) we found more activity in detected than undetected trials, as illustrated in the bar graphs in the difference between the second (167 undetected, light gray) and third bar (167 detected, dark gray), respectively. Thus, BOLD suppression is not only related to small (first bar, light gray, 0-83ms delay) compared to long delays (fourth bar, dark gray, 250-417ms delays), but is also detectable for subjectively undelayed (undetected delay) compared to delayed (detected delay) trials with an identical delay of 167 ms. The white bar illustrates the activity in the passive condition. Brain images illustrate the brain activation for the contrast: 167ms detected>167ms undetected, at p 0.05 uncorrected, inclusively masked for the result pattern observed in the main analysis (Fig. 5). In this analysis all modalities were combined to obtain sufficient detected and undetected trials in the 167 ms condition to perform the analysis. Despite collapsing across modalities/tasks not all experimental runs could be used in 8 of the 19 participants. However, the direction of effects (detected>undetected trial with identical delay) in relevant brain regions, suggests that BOLD suppression is related to the subjective experience, too, and not only to physical delay between action and action consequences.*
